# Nucleus Reuniens Afferents in Hippocampus Modulate CA1 Network Function *via* Monosynaptic Excitation and Polysynaptic Inhibition

**DOI:** 10.3389/fncel.2021.660897

**Published:** 2021-10-12

**Authors:** Priyodarshan Goswamee, Elizabeth Leggett, A. Rory McQuiston

**Affiliations:** Department of Anatomy and Neurobiology, Virginia Commonwealth University School of Medicine, Richmond, VA, United States

**Keywords:** nucleus reuniens, hippocampus, patch-clamp electrophysiology, optogenetics, interneuron-selective interneurons, neurogliaform cells, disynaptic inhibition

## Abstract

The thalamic midline nucleus reuniens modulates hippocampal CA1 and subiculum function *via* dense projections to the stratum lacunosum-moleculare (SLM). Previously, anatomical data has shown that reuniens inputs in the SLM form synapses with dendrites of both CA1 principal cells and inhibitory interneurons. However, the ability of thalamic inputs to excite the CA1 principal cells remains controversial. In addition, nothing is known about the impact of reuniens inputs on diverse subpopulations of interneurons in CA1. Therefore, using whole cell patch-clamp electrophysiology in *ex vivo* hippocampal slices of wild-type and transgenic mice, we measured synaptic responses in different CA1 neuronal subtypes to optogenetic stimulation of reuniens afferents. Our data shows that reuniens inputs mediate both excitation and inhibition of the CA1 principal cells. However, the optogenetic excitation of the reuniens inputs failed to drive action potential firing in the majority of the principal cells. While the excitatory postsynaptic currents were mediated *via* direct monosynaptic activation of the CA1 principal cells, the inhibitory postsynaptic currents were generated polysynaptically *via* activation of local GABAergic interneurons. Moreover, we demonstrate that optogenetic stimulation of reuniens inputs differentially recruit at least two distinct and non-overlapping subpopulations of local GABAergic interneurons in CA1. We show that neurogliaform cells located in SLM, and calretinin-containing interneuron-selective interneurons at the SLM/stratum radiatum border can be excited by stimulation of reuniens inputs. Together, our data demonstrate that optogenetic stimulation of reuniens afferents can mediate excitation, feedforward inhibition, and disinhibition of the postsynaptic CA1 principal cells *via* multiple direct and indirect mechanisms.

## Introduction

The thalamic nucleus reuniens (RE) is source of a major extrinsic glutamatergic input to the hippocampus, along with the medial and lateral entorhinal cortices and basolateral amygdala (Dolleman-Van der Weel et al., 1997; Huff et al., 2016; Li et al., 2017). Previous anatomical tracer studies showed that the RE is reciprocally connected with the hippocampus and medial prefrontal cortex (mPFC) and indicated the existence of a closed loop of information transfer between the mPFC ⇆ RE ⇆ CA1→mPFC (Jay and Witter, 1991; Vertes et al., 2007; Hoover and Vertes, 2012). Consistent with this, RE has been shown to play a critical role underlying a range of hippocampus and mPFC-dependent cognitive processes such as acquisition and recall of spatial working memory (Hallock et al., 2016; Viena et al., 2018; Dolleman-van der Weel et al., 2019), goal-directed spatial navigation (Ito et al., 2015), spatial encoding and head direction (Jankowski et al., 2014) and fear memory (Xu and Sudhof, 2013; Ramanathan et al., 2018).

Reuniens terminals in the hippocampus are known to be largely restricted to the *stratum lacunosum moleculare* (SLM) of CA1 with sparse innervation in the stratum *radiatum* (SR) (Wouterlood et al., 1990). Consistent with the distribution of terminals, direct electrical stimulation of RE was shown to result in a prominent negative field potential or current sink at the SLM (indicative of the location of excitatory synaptic input) and smaller current sources in all other layers of hippocampal CA1 in anesthetized rats (Dolleman-Van der Weel et al., 1997). Notably, the authors reported that stimulation of RE was not able to generate a population spike at the *stratum pyramidale* (SP). However, a subsequent study utilizing similar technique showed that thalamic inputs have comparable ability to excite the CA1 SP as the intrinsic Schaffer collaterals from the contralateral CA3 (Bertram and Zhang, 1999).

It is known that the interneurons in hippocampal CA1 are classified into several distinct subpopulations based on their morphology, gene content, synaptic connectivity and firing properties (Freund and Buzsaki, 1996; Madison and McQuiston, 2006; Klausberger and Somogyi, 2008). Therefore, activation of distinct interneuron subtypes by specific excitatory inputs can give rise to differential effects on network function through the suppression of different components of the hippocampal CA1 network. Ultrastructural evidence showed that the RE inputs in CA1 form synapses with both the spines of the distal apical dendrites of PCs and the aspinous dendrites of interneurons (Dolleman-Van der Weel and Witter, 2000). However, to the best of our knowledge, the impact of glutamate release from the RE inputs on the diverse neuronal subpopulations in CA1 has not been investigated.

To address these gaps in our knowledge, we conducted whole-cell patch clamp electrophysiological measurements from CA1 PCs and interneurons in response to selective optogenetic stimulation of the RE afferents in hippocampal slices obtained from mice. We expressed the excitatory optogenetic protein oChIEF in RE neurons by introducing an adeno-associated virus (AAV) containing the coding sequence of oChIEF-tdtomato under the control of a human synapsin promoter (AAV1-hsyn-oChIEF-tdtomato) (Lin et al., 2009). Our results show that stimulation of RE inputs can give rise to complex modulation of CA1 PC activity *via* multiple cellular and network mechanisms that is largely driven by excitation of distinct subpopulations of interneurons.

## Materials and Methods

### Animals

All transgenic and wildtype C57BL/6 mice used in these studies were housed in a temperature and humidity-controlled vivarium with *ad libitum* access to food and water. The animal care facility is approved by the American Association for the Accreditation of Laboratory Animal Care. Experimental procedures followed the protocol approved by the Institutional Animal Care and Use Committee of Virginia Commonwealth University (AD20205). This protocol adhered to the ethical guidelines described in The Care and Use of Laboratory Animals 8th Edition [National Research Council (U.S.) et al., 2011]. All efforts were made to minimize animal suffering and to reduce the number of animals used. The NPY-cre mice (Jax Stock no. 027851) were sourced from the Jackson laboratories, Bar Harbor, Maine. The CalR; VGAT2AFlp-tdtom mice were generated by the triple crossing of Ai65D (JAX Stock No. 021875) (Madisen et al., 2015), Calb2-IRES-Cre (JAX Stock No. 010774) (Taniguchi et al., 2011) and Slc32a1-2A-FlpO-D knock-in mice (JAX Stock No. 029591) (Gizowski and Bourque, 2020) as per the scheme shown in Figure 7A.

**FIGURE 1:**
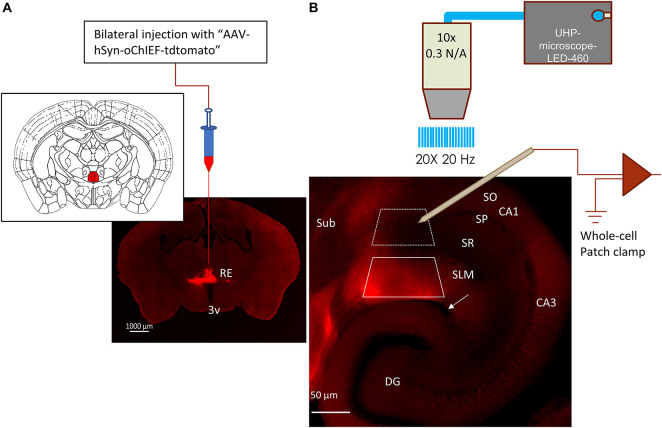
Expression of optogenetic protein in RE inputs to hippocampus. **(A)** Fluorescent image of a coronal brain slice showing the expression of oChIEF-tdtom in the nucleus reuniens (RE) located dorsal to the 3rd ventricle (3v); Inset: corresponding mouse atlas plate showing the site of virus injection (filled red) (Franklin and Paxinos, 2013). **(B)** oChIEF-tdtom expressing RE afferents in hippocampal CA1. The layers are identified as: SO = stratum oriens, SP = stratum pyramidale, SR = stratum radiatum, SLM = stratum lacunosum moleculare, DG = dentate gyrus. The hippocampal fissure is identified by the white arrow. Note that the tdtomato-labeled fibers express almost exclusively in the stratum lacunosum moleculare (SLM) of CA1. Boxes marked with white dashed and solid lines correspond to regions from which principal cells (PCs) or interneurons were sampled, respectively.

**FIGURE 2:**
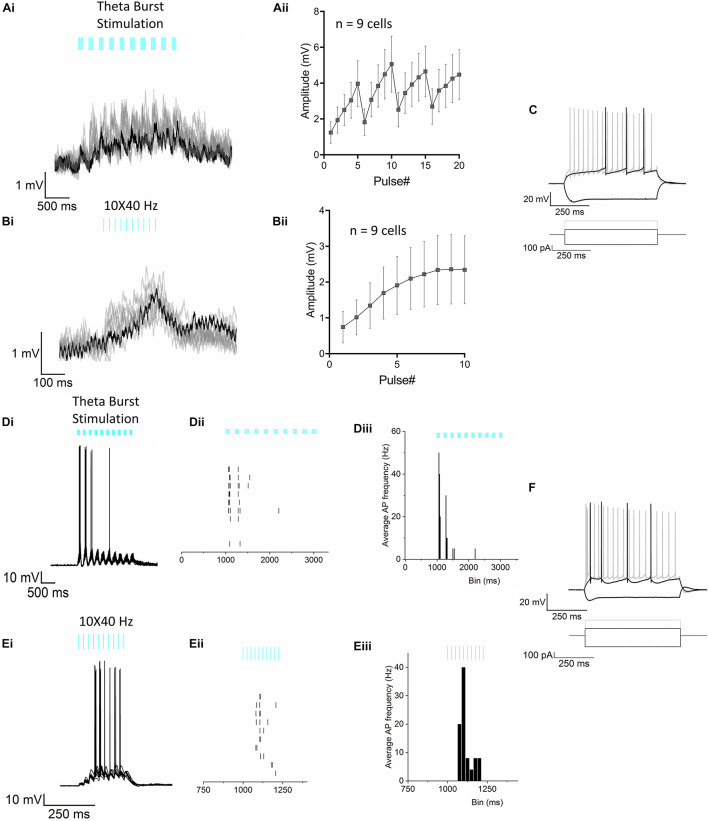
Optogenetic stimulation of RE inputs in CA1 results in mostly subthreshold depolarization of the PCs. **(Ai)** Representative voltage traces (10 trials superimposed) from a CA1 PC in response to the optogenetic TBS paradigm showing subthreshold depolarization of the cell. The TBS paradigm comprised of 10 short bursts of stimulus pulses delivered at a 5 Hz (theta) frequency. Each short train of pulses consisted of 5 pulses at 50 Hz. **(Aii)** Line plot summarizing mean ± S.E.M of membrane potential response to optogenetic stimulation in *n* = 9 cells. **(Bi)** Representative voltage traces (10 trials) from the same neuron shown in panel **(Ai)**, in response to a train of blue light pulses delivered at 10 × 40 Hz showing subthreshold depolarization of the cell. **(Bii)** Line plot summarizing mean ± S.E.M of membrane potential response to optogenetic stimulation in *n* = 9 cells. **(C)** Superimposed traces showing change in membrane potential of the same cell in responses to a series of current steps. Traces shown correspond to injections of hyperpolarizing (–200 pA) and depolarizing currents (rheobase at 100 pA and 2× rheobase 200 pA in gray). **(Di–iii)** AP firing was observed in only 1 neuron. **(Di)** Superimposed traces from 10 consecutive trials from the cell showing AP firing in response to the TBS. **(Dii)** Raster plot showing the temporal relationship of the APs with the light pulses; **(Diii)** Peristimulus time histogram (PSTH) showing the mean instantaneous frequency of AP firing of the neuron. **(Ei–iii)** AP firing in response to the 10 × 40 Hz stimulation paradigm in the same CA1 PC as in panel **(D)**. **(Ei)** Superimposed traces from 10 consecutive trials, **(Eii)** Raster plot of the APs and **(Eiii)** PSTH showing the mean instantaneous frequency of AP firing of the neuron. **(F)** Superimposed traces showing responses to the step protocol correspond to injections of hyperpolarizing (–200 pA) and depolarizing currents (rheobase at 100 pA and 2× rheobase 200 pA in gray).

**FIGURE 3:**
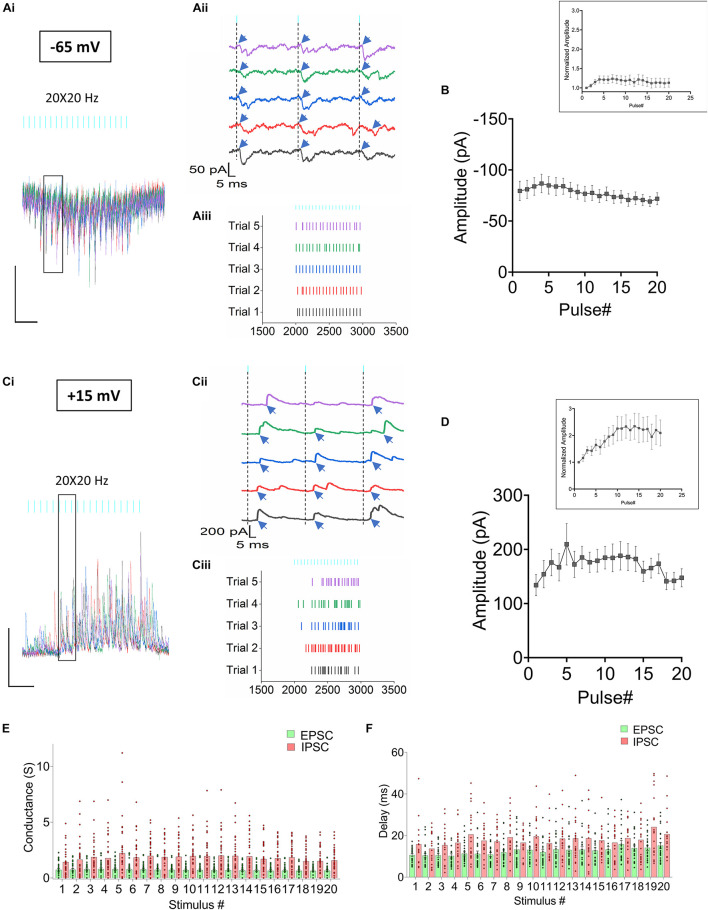
Synaptic current responses from CA1 PCs in response to RE stimulation. **(Ai–iii)** Optogenetic stimulation of RE afferents results in EPSCs **(Ai)** Superimposed traces from 5 consecutive trials obtained from a CA1 PC held at –65 mV. **(Aii)** Magnified view of the individual traces to describe the temporal relationship between the light pulses and the onset of the EPSC events (indicated by blue arrows). **(Aiii)** Raster plot showing distribution of EPSCs in the 5 trials. **(B)** Line plot summarizing the mean ± SEM of EPSC amplitudes measured from 24 CA1 PCs. **(Ci–iii)** Optogenetic stimulation of RE afferents results in IPSCs. **(Ci)** Superimposed traces from 5 consecutive trials obtained from the same CA1 PC as in panel **(A)** held at +15 mV. **(Cii)** Magnified view of the individual traces to describe the temporal relationship between the light pulses and the onset of the IPSC events (indicated by blue arrows). **(Ciii)** Raster plot showing distribution of IPSCs in the 5 trials. **(D)** Line plot summarizing the mean ± SEM of IPSC amplitudes from 28 CA1 PCs. Insets in panels **(B,D)** show the average amplitude responses normalized to the first response. **(E)** Histogram comparing the average conductances of the EPSCs (green) and IPSCs (pink); **(F)** Histogram comparing the delay between the optogenetic pulse and onset of the EPSCs (green) and IPSCs (pink). Individual values are overlaid on the bars in panels **(E,F)** to demonstrate the variability observed in the datasets.

**FIGURE 4:**
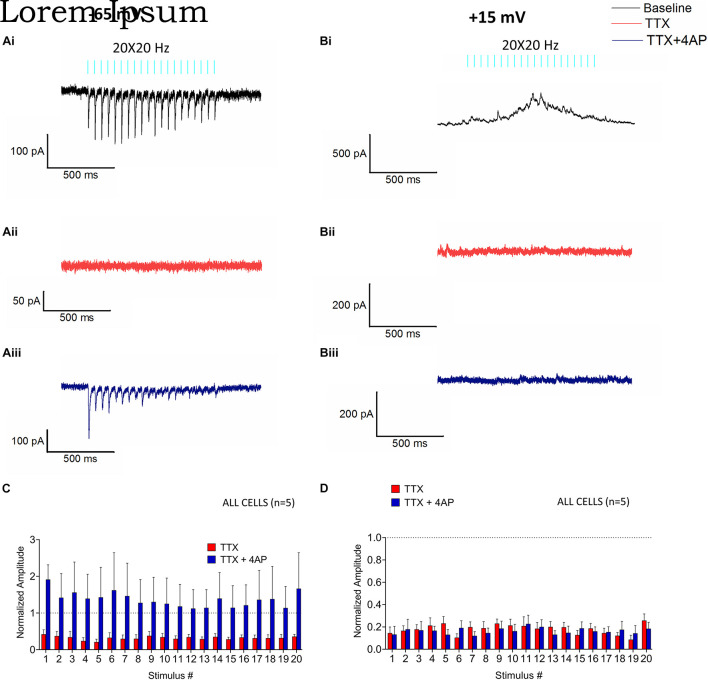
Monosynaptic and polysynaptic effects of RE stimulation on CA1 PCs. Glutamate release from RE inputs mediate monosynaptic EPSCs **(Ai–iii)** and polysynaptic IPSCs **(Bi–iii)**. **(Ai)** Representative trace (black) showing the average baseline EPSC from a recorded cell at –65 mV. **(Aii)** Representative trace (red) showing that bath application of 1 μM tetrodotoxin (TTX) in the same cell completely abolishes the EPSCs. **(Aiii)** Bath application of 4-aminopyridine (4-AP, 100 μM, blue trace) in combination with TTX rescues the EPSCs in the same cell. **(Bi)** Representative trace showing the average baseline IPSC (black) from a recorded cell at +15 mV. **(Bii)** Trace from the same experiment showing that application of TTX abrogates the IPSCs (red). **(Biii)** Bath application of 4-AP in combination with TTX failed to rescue the IPSCs (blue). **(C,D)** Bar plots showing the mean fold change in amplitude of the EPSCs **(C)** or IPSC **(D)** in response to application of TTX (red bars) and TTX in combination with 4-AP (blue bars) (pre-drug baseline indicated by dotted line at *y* = 1). Amplitudes were normalized to the pre-drug baseline and fold-change was analyzed by Holm-Sidak method (one *t*-test/stimulus pulse).

**FIGURE 5:**
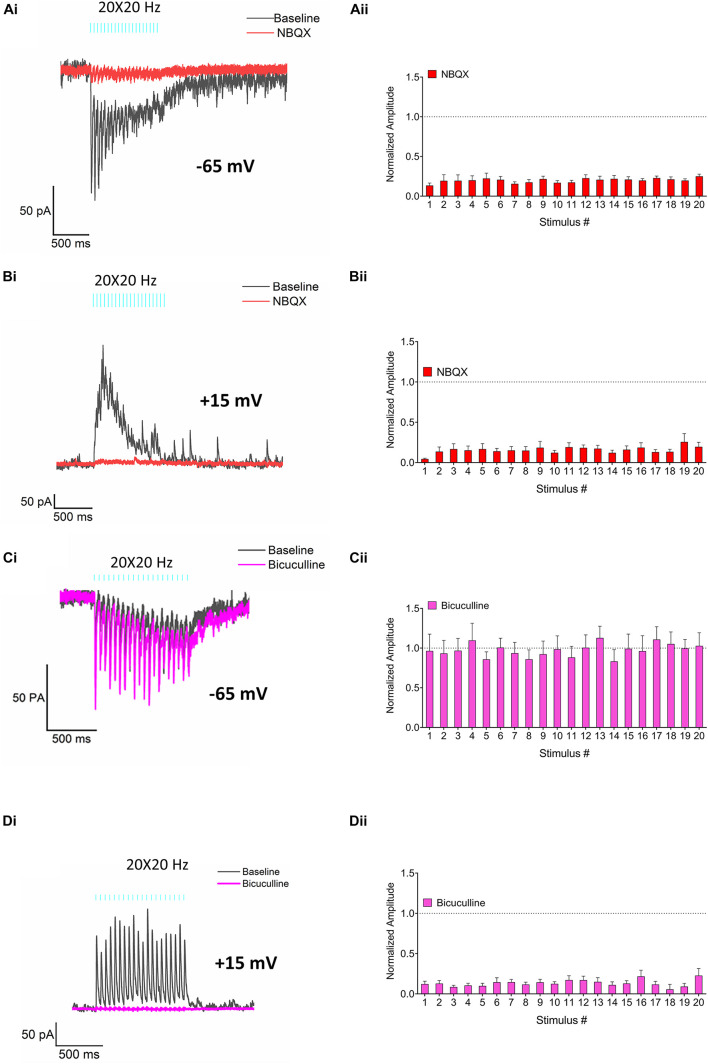
Pharmacological characterization of the EPSCs and IPSCs. **(Ai)** Representative traces demonstrating the effect of the AMPA receptor antagonist NBQX (30 μM, red trace) on RE elicited EPSCs (control, black). **(Aii)** Bar plot–normalized change in EPSC amplitudes produced by NBQX (red bars). **(Bi)** Representative traces demonstrating the effect of NBQX (30 μM, red trace) on IPSCs (control, black). **(Bii)** Bar plot–normalized change in IPSC amplitudes in response to application of NBQX (red bars). **(Ci)** Representative traces showing the effect of bicuculline (25 μM, pink trace) on EPSCs (control, black). **(Cii)** Bar plot -normalized change in EPSC amplitudes in response to application of bicuculline (pink bars). **(Di)** Representative traces demonstrating the effect of bicuculline (25 μM, pink trace) on IPSCs (control, black). **(Dii)** Bar plot–normalized change in IPSC amplitudes in response to application of bicuculline (pink bars). Amplitudes in panels **(Aii,Bii,Cii,Dii)** were normalized to control and fractional change was analyzed by Holm-Sidak method (one *t*-test/stimulus pulse).

**FIGURE 6:**
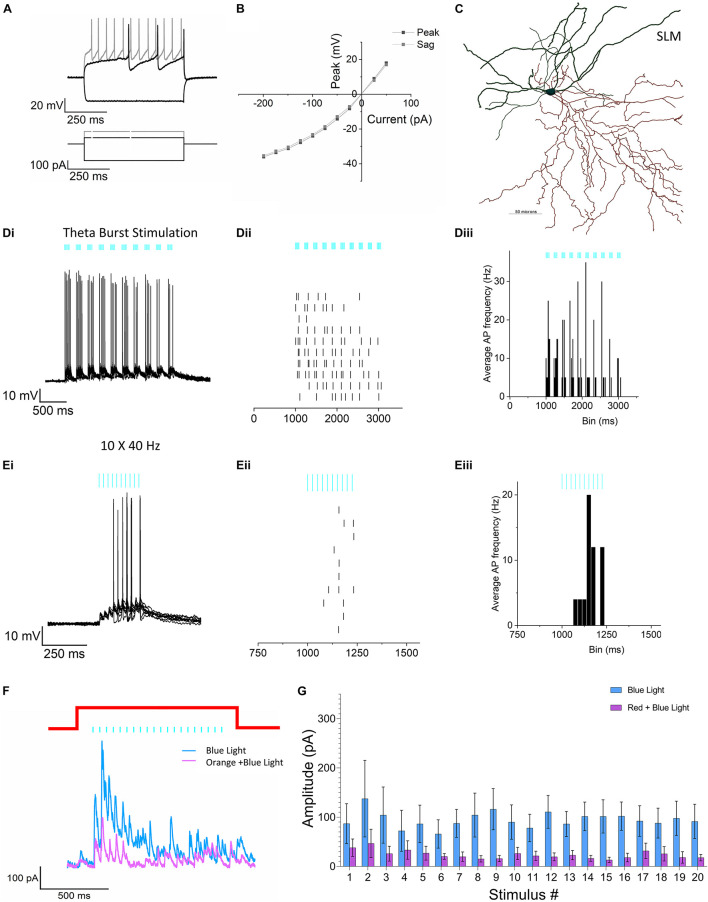
Effect of RE stimulation on NGF interneurons. **(A)** Membrane potential responses to injection of hyperpolarizing (–200 pA) and depolarizing currents (rheobase at 75 pA and 2× rheobase 150 pA, gray). **(B)** Line plot showing current-voltage relationship of the same cell in panel **(A)** in response to a series of hyperpolarizing current steps. **(C)** Morphological reconstruction of an NGF cell located in the SLM of CA1. Scale bar = 50 μm. **(Di)** Superimposed traces showing action potential firing from 10 consecutive trials recorded from an NGF cell in response to TBS. **(Dii)** Raster plot showing the timing of APs in each trial. **(Diii)** Peristimulus time histogram (50 ms bins) showing normalized average AP frequency. **(Ei)** Superimposed traces showing action potential firing from 10 consecutive trials recorded from the same cell in response to a train of 10 pulses at 40 Hz (10 × 40 Hz). **(Eii)** Raster plot showing the timing of APs in each trial. **(Eiii)** Peristimulus time histogram (50 ms bins) showing normalized average instantaneous frequency of AP firing. **(F)** Representative traces showing IPSC at baseline (blue trace) and following suppression of the NPY cells (pink trace) from a CA1 PC. **(G)** Histogram showing summary data of IPSCs from *n* = 7 cells from 2 mice. Data was analyzed using a repeated measures two-way ANOVA.

**FIGURE 7:**
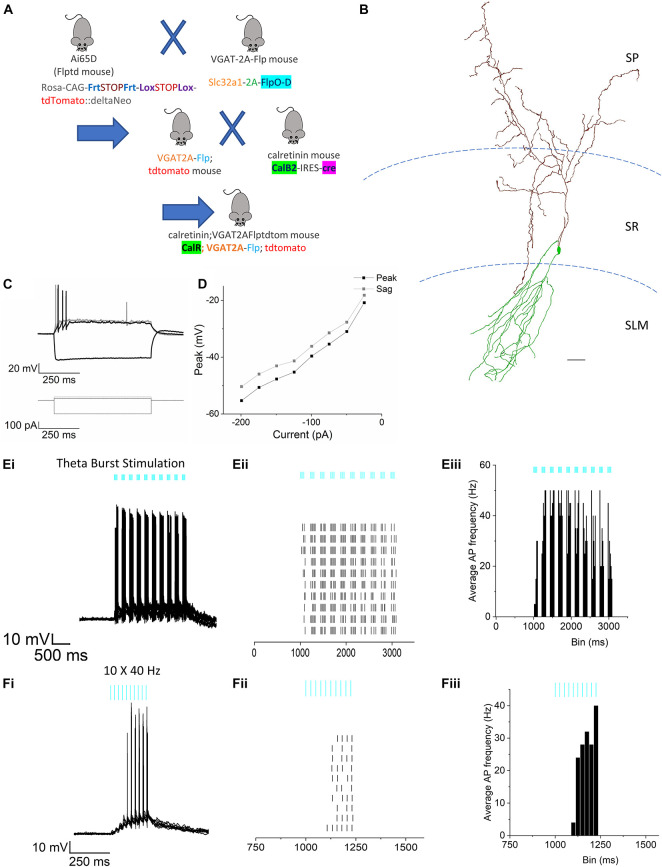
Effect of RE stimulation on calretinin+ IS-interneurons. **(A)** Schematic illustration showing breeding strategy to obtain fluorescent expression of tdTomato in calretinin interneurons. **(B)** Morphological reconstruction of a calretinin cell located at the border of SR and SLM in CA1. Scale bar = 200 μm. **(C)** Membrane potential responses to hyperpolarizing (–200 pA) and depolarizing current injections (25 and 50 pA) of the same cell shown in panel **(B)**. **(D)** Line plots showing current-voltage relationship in response to injection of a series of hyperpolarizing current steps. **(Ei–iii)** Representative example of a calretinin cell response to TBS. **(Ei)** Superimposed traces showing action potential firing from 10 consecutive trials. **(Eii)** Raster plot showing the timing of APs in each trial. **(Eiii)** Peristimulus time histogram (50 ms bins) of the normalized average AP frequency. **(Fi–iii)** Representative example of the response from the same cell as (E) to the 10 × 40 Hz stimulation. **(Fi)** Superimposed traces showing action potential firing from 10 consecutive trials. **(Fii)** Raster plot showing the timing of APs in each trial. **(Fiii)** Peristimulus time histogram (50 ms bins) of the normalized average AP frequency.

### Stereotaxic Surgery

To express oChIEF-tdtomato in the RE of mice, a recombinant adeno-associated virus (rAAV, serotype 1, 4.8 × 10^13^ VC/ml titer) expressing oChIEF-tdtomato [Addgene #50977 packaged by Vector Biolabs (Malvern, PA, United States)] was introduced in the RE. Because of the close proximity of the RE and intermediate hippocampus, it was not possible to document or evaluate expression of the oChIEF at the site of injection in animals utilized for physiology. However, optimization of the stereotaxic surgery to ensure targeting of RE was carried out in a separate pilot study conducted previously in the lab. Briefly, mice (6 weeks–5 months old) were initially anesthetized *via* intraperitoneal injection of ketamine (100 mg/kg IP) and xylazine (2.5 mg/kg IP). Anesthesia was maintained with O_2_ supplemented with 1.0% isoflurane. For injections into the RE, an incision was made in the skin along the mid-sagittal suture, and a small hole was drilled in the skull overlying the RE. An aluminosilicate glass pipette containing rAAV-hSyn-oChIEF-tdtomato was lowered to the level of the RE and infused at a rate of 100 nl/min using a software-driven injectomate (Neurostar, Sindelfingen, Germany). In total, 4 nl × 100 nl injections into the RE were made at AP = 0.7 mm and 0.9 mm caudal to Bregma, ML = ± 0.2 mm, and DV 4.0 mm. Mice were sacrificed 3–5 weeks after surgery to prepare hippocampus slices for electrophysiology. In some experiments, an additional AAV construct encoding the inhibitory optogenetic protein Jaws (AAV5-CAG-FLEX-JAWS-KCG-GFP-ER2) was introduced bilaterally in the SLM region of CA1 using the coordinates (in mm) AP = −3.2; ML = ± 3.45; DV = 3.0–4.0. A total of 4 injections (50 nl each) were made on either side of the brain.

### Preparation of Hippocampal Slices

Brain slices were obtained by methods previously described (Goswamee and McQuiston, 2019). Mice were anesthetized with an intraperitoneal injection of ketamine (200 mg/kg) and xylazine (20 mg/kg). Mice were transcardially perfused with ice cold sucrose-saline [consisting of (in mM): Sucrose 230, KCl 2.5, CaCl_2_ 2, MgCl_2_ 6, NaHPO_4_ 1, NaHCO_3_ 25, glucose 25] and sacrificed by decapitation. The brain was removed and hemi-sected, and horizontal slices containing the mid temporal hippocampus were cut at 350 μm on a Leica VT1200 (Leica Microsystems, Buffalo Grove, IL, United States). Sections were incubated in a holding chamber kept at 32°C. The holding chamber solution consisted of artificial cerebrospinal fluid (aCSF) (in mM): NaCl 125, KCl 3.0, CaCl_2_ 1.2, MgCl_2_ 1.2, NaHPO_4_ 1.2, NaHCO_3_ 25, glucose 25 bubbled with 95% O_2_ / 5% CO_2_. Recordings were performed at 32–35°C.

### Light-Evoked Activation of oChIEF-Expressing RE Inputs and Jaws Expressing NPY Cells in Hippocampal CA1

Reuniens terminals expressing oChIEF-tdtomato were stimulated by a train of 20 blue light pulses (1 ms in duration) delivered at 20 Hz frequency (20 × 20 Hz protocol). The light pulses were generated from a light-emitting diode (LED) (UHP-microscope-LED-460, Prizmatix Modiin Ilite, Givat Shmuel, Israel). Blue light exiting the LED was reflected by a dichroic mirror (515dcxru, Chroma Technology, Bellows Falls, VT, United States) using an optiblock beam combiner (Prizmatix) and focused into the epi-illumination light path of an Olympus BX51WI microscope and back aperture of a 10× water immersion objective (0.3 NA) by a dichroic mirror (700dcxxr, Chroma Technology, Bellows Falls, VT, United States) in the filter turret. The optogenetic parameters were kept constant for all experiments. To excite Jaws, an orange-light pulse was generated using the UHP-TLED-White light-emitting diode (LED) (Prizmatix Modiin-Ilite, Givat Shmuel, Israel). The white light exiting the LED was filtered by a HQ 605/50× filter (Chroma Technology).

### Electrophysiological Measurements

Whole cell patch clamp recordings from hippocampal CA1 interneurons were performed using patch pipettes (2–4 MΩ) pulled from borosilicate glass (8,250 1.65/1.0 mm) on a Sutter P-1000 pipette puller and were filled with intracellular recording solution that contained either a potassium-based recording solution [(in mM): KMeSO_4_ 145, NaCl 8, Mg-ATP 2, Na-GTP 0.1, HEPES 10, EGTA 0.1] or a cesium-based recording solution [(in mM): CsMeSO_4_ 120, NaCl 8, Mg-ATP 2, Na-GTP 0.1, HEPES 10, Cs-BAPTA 10, QX-314 Chloride 10]. In a subset of experiments, 0.2% biocytin was included in the intracellular recording solution for *post hoc* evaluation of morphology of the recorded cell. Membrane potentials and/or currents were measured with a Model 2400 patch clamp amplifier (A-M Systems, Port Angeles, WA, United States) and converted into a digital signal by a PCI-6040E A/D board (National instruments, Austin, TX, United States). Voltage clamp experiments where the access resistance changed by more than approximately 20% were discarded. WCP Strathclyde Software was used to store and analyze membrane potential and current responses on a PC (courtesy of Dr. J Dempster, Strathclyde University, Glasgow, Scotland). Calculated junction potentials (9.4 and 10 mV, respectively) were not compensated for in the analysis. Further analysis was performed with OriginPro 2018 (OriginLab Corp., Northampton, MA, United States) and Graphpad Prism (San Diego, CA, United States).

### Morphological Evaluation of Interneurons

Following electrophysiological recordings, slices were fixed in 4% paraformaldehyde (Boston Bioproducts) and incubated with streptavidin Alexa Fluor 633 (ThermoFisher Scientific, Waltham, MA, United States) in phosphate buffered saline (PBS) with Triton-X 100 as previously described (Bell et al., 2011). Processed slices were then imaged using a Zeiss LSM 710 confocal microscope (Carl Zeiss, Jena, Germany). Alexa Fluor 633 was excited with the 633 nm line of a HeNe 5 mW laser and cells were visualized using a 20× dry lens (0.8 N.A., voxel dimensions 0.2 μm × 0.2 μm × 1.1 μm). In some cases, the Z-stacks were processed using the Neurolucida 360 software for morphological reconstruction of the filled cell.

### Statistics and Data Analysis

Data were analyzed using WCP software and OriginPro 2018 for the electrophysiological measurements. Statistics were performed using GraphPad Prism (San Diego, CA, United States). Statistical significance for facilitation in amplitude of postsynaptic potentials and currents recorded from CA1 PCs and interneurons was determined by normalizing the amplitudes of the responses of the first pulse. The slope of the facilitation was determined by performing a test of simple linear regression of the normalized data. Statistical significance of comparison of the conductances underlying the excitatory postsynaptic currents (EPSC) and inhibitory postsynaptic currents (IPSC) was determined by repeated measures two-way ANOVA. Conductance was determined by dividing the current (I) by the difference between the holding potential (E_hold_) and the reversal potential for the channel (E_rev_): $\text{Conductance}\,\left(\text{g}\right)=\left|\frac{\text{I}}{{\text{E}}_{\text{hold}}-{\text{E}}_{\text{rev}}}\right|$. A repeated measures 2-way ANOVA was utilized to compare the ionic conductances at −65 mV and +15 mV. To identify EPSC and IPSC events and determine the latency of the events, five consecutive trials of every cell recorded were analyzed using the threshold search algorithm of the open-sourced analysis package (easy electrophysiology V2.2.0). For the event search, a 15 ms baseline was set, and the onset of an event was defined as 5% of the maximum amplitude. To calculate the latency, the onset time of an event was subtracted from the onset of optogenetic pulse in individual events. In cases where events followed a compound waveform or had multiple peaks, the first peak was utilized for analysis of the latency. The kinetic properties of the EPSCs and IPSCs were compared using a mixed-model ANOVA. To evaluate the effects of pharmacological agents (Figures 4, 5), on the EPSCs and IPSCs, the average “post-drug” current amplitudes were normalized to their respective “pre-drug” or “baseline” averages. This treatment of the data resulted in a lack of variance in the pre-drug data; thus, the statistical significance of the effects of the drugs was determined by using multiple *t*-tests (one for each stimulus number). Statistical differences were determined using the Holm-Sidak method with alpha = 0.05. The range of *P* values are provided in text. Comparison of the mean action potential firing frequencies between neurogliaform cells and calretinin cells in Figure 7 was performed by an unpaired *t*-test. All data was reported as the mean and standard error of mean (SEM). Asterisks were as follows: ^∗∗∗^*P* < 0.001, ^∗∗^*P* < 0.01, and ^∗^*P* < 0.05.

### Chemicals

All chemicals were purchased from VWR unless otherwise indicated. QX314 chloride and 4-aminopyridine (4-AP) were purchased from Sigma-Aldrich, (St. Louis, MO, United States). Tetrodotoxin citrate (TTX), bicuculline methochloride, 2,3-Dioxo-6-nitro-1,2,3,4-tetrahydrobenzo[f]quinoxaline-7-sulfonamide disodium salt (NBQX), and DL-2-Amino-5-phosphono pentanoic acid (APV) were purchased from Hello Bio (Princeton, NJ, United States). Biocytin (B-1592) and streptavidin Alexa 633 were purchased from ThermoFisher Scientific.

## Results

### Optogenetic Stimulation of Reuniens Inputs Resulted in Subthreshold Stimulation of CA1 PCs

Optogenetic stimulation of RE terminals was achieved by AAV-mediated expression of the excitatory optogenetic protein oChIEF-tdtomato in RE neurons (Figure 1A). We observed RE afferents in CA1 and the subiculum of the hippocampus (Figure 1B). In CA1, RE afferents remained largely restricted to the SLM layer with very few terminals extending to the SR. In contrast, in the subiculum, the RE afferents could be observed in all layers. To determine the effect of RE stimulation on the membrane potential of CA1 PCs, we optogenetically activated RE afferents in hippocampal slices using physiologically relevant frequency bands such as theta (5 Hz) and gamma (40 Hz). Because RE neurons are known to fire both tonically as well as in bursts (Zimmerman and Grace, 2018), we stimulated the oChIEF expressing RE inputs using brief blue light flashes (460 nm, 1 ms) delivered using either theta-burst (50 Hz bursts delivered at 5 Hz frequency) or gamma (10 × 40 Hz) frequency. Light-evoked changes in the postsynaptic membrane potential in CA1 PCs were initially measured in the current clamp mode by clamping the CA1 PCs at −65 mV. As shown in Figures 2A,B, optogenetic stimulation of the RE afferents using the theta-burst stimulation (TBS) or the 10 × 40 Hz paradigm resulted in small, summating EPSPs in 9 out of 10 cells recorded (3 mice, 1 cell/slice). The trials (8–10 trials/cell) were averaged, and the baseline subtracted to determine the amplitude of the EPSP responses. The average maximum amplitudes of the depolarization using TBS and the 10 × 40 Hz paradigm were 5.062 ± 1.568 mV and 2.358 ± 0.969 mV, respectively. However, action potential (AP) firing was noted in one CA1 PC (Figures 2D,E). Furthermore, all cells had passive membrane and intrinsic firing properties consistent with CA1 PCs (Figures 2C,F). Therefore, these data indicate that stimulation of RE afferents in the hippocampus resulted in AP firing in only a small subset of PCs. However *in vivo* stimulation of nucleus reuniens fiber onto CA1 PCs may produce a different outcome. In a separate set of experiments, we determined that the short-term kinetics of glutamate release was comparable in response to optogenetic or electrical stimulation of the CA3 Schaffer collateral pathway (Supplementary Figure 1).

### Optogenetic Stimulation of Reuniens Inputs Generated Both Excitatory and Inhibitory Postsynaptic Currents in CA1 PCs

Next, we conducted voltage clamp experiments utilizing a Cs^+^-based pipette solution to record both excitatory (EPSC) and inhibitory (IPSC) postsynaptic currents from CA1 PCs. To measure light-evoked EPSCs (Figure 3A), PCs were held at −65 mV near the equilibrium potential for chloride. To measure IPSCs, PCs were held near the reversal potential for glutamate, empirically determined to be approximately +15 mV (IPSCs, Figure 3C). A total of 33 CA1 PCs responded to optogenetic stimulation, of which 19 cells responded with both EPSCs and IPSCs, 5 neurons presented with EPSCs, but no IPSCs. 9 neurons responded with IPSCs, but no detectable EPSC. Data were pooled for analysis. The EPSCs were small (Figure 3B average maximum amplitude was −86.8 ± 8.95 pA, *n* = 24). In comparison to the current clamp data, the EPSCs showed less facilitation when the responses were normalized to the amplitude of the first response (Figure 3B, inset; slope = 0.0002066, simple linear regression). The amplitude of the IPSCs were often larger but more variable (Figure 3D, average maximum amplitude was 209.63 ± 38.31 pA, *n* = 28).

Because ionic driving forces at these two holding potentials (−65 mV and +15 mV) were different, we compared the conductances to determine the relative strengths of inhibition vs excitation. A repeated measures 2-way ANOVA showed that the inhibitory conductances were significantly larger than the excitatory conductances (Figure 3E, repeated measures 2-way ANOVA, *P* < 0.0001, *n* = 33). We further compared the synaptic delay between glutamate release (light pulses) and the onset of the EPSC and IPSC events. Analysis of the current traces from neurons that responded to optogenetic stimulation showed that latency between the light pulses and the onset of events was significantly shorter for the EPSCs (12.5 ± 0.3018 ms; *n* = 24) compared to the IPSCs (18.05 ± 0.4668 ms; *n* = 29) (Figure 3F, mixed-model ANOVA, *P* < 0.0001). Therefore, optogenetic stimulation of RE inputs in CA1 resulted in both excitation and inhibition of the CA1 PCs.

### Stimulation of Reuniens Afferents Drive Feed-Forward Inhibition *via* a Polysynaptic Mechanism

Next, we utilized pharmacological tools to further investigate neural network mechanisms underlying optogenetically-elicited EPSCs and IPSCs. To examine the possible monosynaptic nature of postsynaptic responses, we bath-applied the voltage-dependent sodium channel antagonist 1 μM tetrodotoxin (TTX), which results in inhibition of action potential generation and action potential-dependent release of neurotransmitter. TTX application inhibited both EPSCs (Figure 4Aii, red trace, *t*-test; 0.000003 ≤ *p* ≥ 0.003164) and IPSCs (Figure 4Bii, red trace, *t*-test *p* < 0.0001). This demonstrated that EPSCs and IPSCs required action potential dependent release of neurotransmitter. The potassium channel antagonist 4-aminopyridine (4-AP) has been previously shown to rescue TTX inhibited monosynaptic connections presumably by permitting larger depolarizations of synaptic terminals, calcium influx, and the release of neurotransmitter (Petreanu et al., 2007). When we applied 100 μM 4-AP in an attempt to rescue TTX inhibition of EPSCs and IPSCs, 4-AP rescued the EPSCs (Figure 4Aiii, 0.05212 ≤ *p* ≥ 0.8194), but not IPSCs (Figure 4Biii, *p* < 0.0001). These data suggest that EPSCs were generated *via* monosynaptic excitation of the CA1 PCs, whereas the IPSCs were generated *via* a polysynaptic pathway.

We next examined the transmitters and receptors responsible for the EPSCs and IPSCs generated by RE inputs in CA1 PCs. Bath application of NBQX, a selective inhibitor of AMPA receptors, suppressed both EPSCs (red trace, Figures 5Ai,ii, multiple *t*-tests, *p* < 0.0001; *n* = 6) and IPSCs (red trace, Figures 5Bi,ii, multiple *t*-tests, *p* < 0.0001; *n* = 6) consistent with a monosynaptic excitatory connection and polysynaptic inhibition. IPSCs were also blocked by the GABA_A_ receptor inhibitor bicuculline (Figures 5Di,ii, pink traces, multiple *t*-tests, *p* < 0.0001; *n* = 8) whereas the EPSCs were unaffected (Figures 5Ci,ii, pink traces, multiple *t*-tests, 0.1593 ≤ *p* ≥ 0.9791; *n* = 7). Therefore, monosynaptic glutamate release from the RE inputs resulted in AMPA receptor mediated EPSCs whereas polysynaptic IPSCs resulted from the activation of GABA_A_ receptors.

### Optogenetic Stimulation of Reuniens Afferents Can Recruit NGF Cells in Stratum Lacunosum Moleculare

To probe the origin of the inhibitory conductances, we performed current clamp recordings from interneurons present within the RE terminal field in SLM. Biocytin (0.2%) was included in the pipette solution for *post hoc* morphological identification of the interneurons from which we recorded. Neurons were recorded from in current-clamp mode and depolarizing and hyperpolarizing current steps were injected to determine their passive membrane and firing properties (summarized in Table 1). These neurons had mean resting membrane potentials of −62.82 ± 1.03 mV, displayed little to no depolarizing sag in the membrane potential in response to a series of hyperpolarizing pulses (Figures 6A,B). In response to rheobase depolarizing pulses, most of these cells showed a delay to the generation of APs (105.7 ± 28.37 ms from the onset of the depolarizing pulse) and the APs were typically followed by a large afterhyperpolarization (AHP). Morphological evaluation of the filled neurons revealed that these neurons have a spherical soma surrounded by short dendritic arborization. The axons, when visible, formed dense plexus confined to the SLM (Figure 6C). The passive membrane and firing properties and morphological features of these neurons are consistent with those described for neurogliaform (NGF) neurons. (Williams et al., 1994; Overstreet-Wadiche and McBain, 2015).

**TABLE 1:** Electrophysiological properties of known cell types recorded in this study.

Cell Type	Resting Membrane Potential (mV)	Input Resistance (mΩ)	I_h_ Sag Ratio	AP Amplitude (mV)	AP threshold (mV)	AP duration (ms)	AP mean frequency (Hz)	Rheobase (pA)	Latency to 1st AP (ms) at Rheobase
CA1 PC (*n* = 9)	−64.67 ± 0.4714	167.9 ± 26.50	0.7898 ± 0.02774	96.02 ± 5.559	−55.45 ± 2.630	1.060 ± 0.1336	39.09 ± 20.01	109.1 ± 8.210	115.2 ± 34.37
NGF cells (*n* = 10)	−64.10 ± 1.120	332.8 ± 30.28	0.9583 ± 0.01	86.32 ± 3.785	−58.68 ± 2.712	1.050 ± 0.102	65.67 ± 28.85	96.69 ± 14.95	165.4 ± 40.52
Calretinin ISIs (*n* = 10)	−63.30 ± 1.291	696.4 ± 104.0	0.9512 ± 0.008	78.35 ± 6.982	−51.25 ± 1.739	0.7100 ± 0.1001	64.28 ± 19.84	49.69 ± 6.435	40.85 ± 11.16

Optogenetic stimulation of RE inputs resulted in AP firing in 5 out of 10 cells in response to TBS (Figure 6D) and 4 out of 10 cells in response to the 10 × 40 Hz stimulation paradigm (Figure 6E). Together these data suggest that physiological relevant stimulation of RE inputs were capable of activating NGF interneurons.

The majority of NGF cells in the SLM of hippocampal CA1 express NPY (Price et al., 2005). Therefore, we utilized a transgenic mouse line in which Cre recombinase is expressed in neuropeptide Y neurons (NPY-c) in order to examine the potential role that NGF neurons play in mediating feed-forward inhibition by RE inputs. To do this, NPY-c mice were intracranially injected bilaterally in the SLM of hippocampal CA1 with an AAV that required Cre-recombinase for expression of the inhibitory optogenetic protein Jaws (Chuong et al., 2014). This permitted the selective expression of Jaws in NPY neurons. The same animals also received an injection of AAV expressing the excitatory optogenetic protein oChIEF in the RE. We subsequently recorded polysynaptic IPSCs from CA1 PCs in *ex vivo* brain slices, as described in the previous section. Polysynaptic IPSCs driven by RE inputs were elicited by a 20 pulse 20 Hz train of blue light pulses. On alternating trials, NPY interneurons were inhibited by a 1,400 ms orange-light pulse during the activation of RE inputs by blue light pulses (Figure 6F). Comparison of the amplitude of the polysynaptic IPSCs in response to the 20 Hz blue light train with and without the orange-light flash showed that activation of Jaws resulted in significant reduction in the amplitude of the polysynaptic IPSCs (Figure 6G; repeated-measures 2-way ANOVA, *P* = 0.027; *n* = 7 cells from 2 mice). This suggests that NPY interneurons (likely NGF interneurons) play a significant role in mediating feed-forward inhibition of CA1 PCs by RE inputs.

### Optogenetic Stimulation of Reuniens Afferents Activates Interneuron-Selective Interneurons at the Stratum Lacunosum Moleculare/Stratum Radiatum Border

**FIGURE 8:**
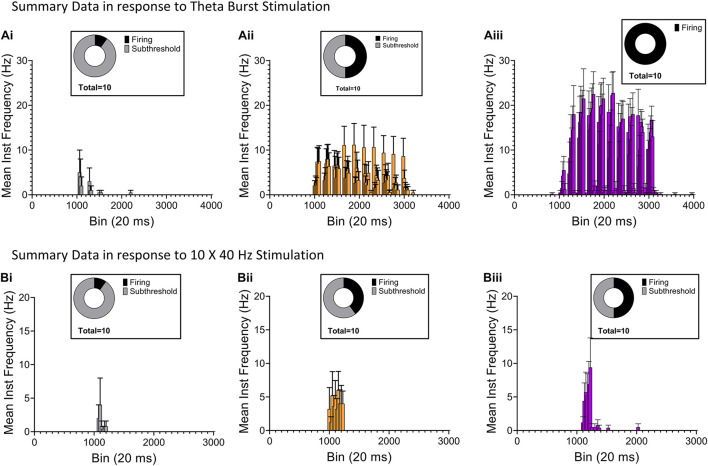
Comparison of AP firing in CA1 PC, NGF and calretinin (+) IS-interneurons elicited by RE synaptic inputs. **(Ai–iii)** Histograms summarizing average instantaneous frequency of AP firing in response to TBS from 10 CA1 PCs [**(Ai)**, gray], 10 NGF cells [**(Aii)**, orange], and 10 calretinin (+) IS-interneurons [**(Aiii)**, purple]. **(Bi–iii)** Histograms summarizing average instantaneous frequency of AP firing in response to 10 × 40 Hz stimulation from 10 CA1 PCs [**(Bi)**, gray], 10 NGF cells [**(Bii)**, orange], and 10 calretinin (+) IS-interneurons [**(Biii)**, purple]. Insets in every panel shows pie-chart summarizing the distribution of firing (black) and subthreshold (gray) responses from the respective data-set.

Next, we examined the effect of RE input optogenetic stimulation on the interneuron-selective (IS) interneurons at the SR/SLM border in CA1 (Type 2 ISIs) (Pelkey et al., 2017). This subpopulation of interneuron is known to express the calcium binding protein calretinin (Gulyás et al., 1996). These specialized interneurons are known to synapse and inhibit other interneurons resulting in disinhibition of CA1 PCs. To identify these interneurons in our hippocampal slice preparation, we triple crossed the Ai65D line (Madisen et al., 2015) with the VGAT-2A-Flp line (Gizowski and Bourque, 2020) and the calretinin-cre line (Taniguchi et al., 2011; Figure 7A). This cross resulted in the selective expression of tdTomato in calretinin interneurons in hippocampal CA1. We used this cross to target recordings from calretinin interneurons with somas located at the SR/SLM border. These cells typically had very small, fusiform somas with extensive dendritic arbor in the SLM and axons extending into the SP and SR (example in Figure 7B). As shown in Figures 7C,D, like the NGF neurons, these neurons display a modest sag in response to injection of negative current (Figure 7A). Optogenetic stimulation of the RE inputs utilizing the TBS paradigm resulted in high frequency AP firing in all cells recorded (Figure 7E). However, when the same neurons were subjected to the 10 × 40 Hz paradigm, only 6 out of 10 cells responded with AP firing (Figure 7F). The complete summary data for the average instantaneous frequency for all the cells in response to TBS and 10 × 40 Hz paradigm are shown in Figures 8Aii,Bii, respectively. Averaging the peristimulus time histogram data from all neurons that responded with AP firing revealed that the maximum AP firing frequency of NGF neurons in response to TBS and 10 × 40 Hz paradigm were 11.11 ± 4.214 Hz and 6.00 ± 2.813 Hz, respectively. In contrast, the calretinin-containing IS interneurons responded to TBS and 10 × 40 Hz stimulation with maximum AP firing frequencies of 22.75 ± 4.779 Hz and 9.4 ± 4.4 Hz, respectively (Figures 8Aiii,Biii). The CA1 PCs were least excited by optogenetic stimulation of RE inputs and the TBS and 10 × 40 Hz paradigms; the maximum AP firing frequency was 5.00 ± 5.000 Hz and 4.00 ± 4.00 Hz, respectively (Figures 8Ai,Bi). To enable comparison of the excitability of the three cell types, CA1 PCs were sampled from the same mice (and often from the same slice) from which the interneurons were sampled. This comparison revealed that in response to optogenetic stimulation of RE inputs, the calretinin interneurons showed the highest degree of excitation, while CA1 PCs showed the least excitation both in terms of proportion of cells that showed AP firing as well as average maximum instantaneous frequencies.

## Discussion

In the present manuscript, we examine the physiological consequences of RE inputs in CA1. We show that RE inputs provide a direct, albeit weak monosynaptic excitation as well as a more robust feedforward inhibition *via* excitation of CA1 interneurons. Further, we demonstrate the impact of RE stimulation in two distinct subpopulations of CA1 interneurons —NGF cells and calretinin-containing interneuron-selective interneurons. Thus, our studies provide evidence that support RE-mediated excitation, feed-forward inhibition and disinhibition of CA1 PCs *via* activation of specific subpopulations of CA1 interneurons.

Previous studies have reported the effect of RE input on population activity in CA1 of anesthetized rats (Dolleman-Van der Weel et al., 1997; Bertram and Zhang, 1999). While one study reported that electrical stimulation of RE failed to evoke a population spike in CA1 (Dolleman-Van der Weel et al., 1997), another group reported RE stimulation could evoke CA1 population spikes (Bertram and Zhang, 1999). These differences may be due to the differences between stimulation paradigms in these two studies. Moreover, there remains the possibility that the population spike may have been driven by antidromic spikes elicited by stronger stimulation of CA1 PC terminals, which project to RE. Nevertheless, our results agree with both *in vivo* studies, which suggest that RE inputs directly excite CA1 distal apical dendrites through monosynaptic connections. However, we also measured inhibitory responses in CA1 PCs that were mediated by a polysynaptic network comprised of inhibitory interneurons in the SLM. This conclusion was supported by the observations that the EPSCs displayed shorter latency than the IPSCs. In addition, pharmacological inhibition of AMPA receptors resulted in suppression of both EPSCs and IPSCs. Finally, pharmacological recovery of neurotransmitter release *via* application of 4-AP (in presence of TTX) could rescue the EPSCs but not IPSCs. Taken together, these observations suggest that the RE has direct excitatory input onto CA1 PCs but requires polysynaptic activation to produce IPSCs (Petreanu et al., 2007).

Our data have demonstrated that trains of optogenetic stimulation of RE terminals resulted in facilitating EPSPs in CA1 PCs and interneurons, suggesting low probability of release synapses and is consistent with paired-pulse data from previous field potential studies (Dolleman-Van der Weel et al., 1997). Interestingly, the current traces obtained at −65 mV from voltage clamp experiments in PCs appeared to produce less facilitation. This would indicate that when stimulated at 20 Hz, the release of vesicles between successive pulses from the RE inputs remained relatively uniform, but the effect of glutamate release on the depolarization of the postsynaptic neuron resulted in summation. Thus, the facilitation observed in the current clamp experiment and previous field potential experiments (Dolleman-Van der Weel et al., 1997) may be due to the activation of voltage-dependent ion channels or nonlinear electrical properties of the dendritic membrane, leading to the summation of EPSPs in successive pulses of the stimulus train and not due to presynaptic release mechanisms.

Fifty percent of RE afferents form synaptic connections with aspinous dendrites of local GABAergic interneurons in the SLM (Dolleman-Van der Weel and Witter, 2000). However, physiological data demonstrating recruitment of these cells by the RE has been lacking. In the present studies, we compared the effect of RE stimulation on two different non-overlapping subpopulations of interneurons present in the RE terminal field. The largest population of interneurons located in the SLM are the NGF cells (Lacaille and Schwartzkroin, 1988; Williams et al., 1994; Price et al., 2005; Capogna, 2011; Overstreet-Wadiche and McBain, 2015). These cells are characterized by their spherical or fusiform soma with dense dendritic arborization that remain confined to the SLM. Axonal branches are typically short and local; however, some have been reported to reach SR of CA3, as well as to the molecular layer in dentate gyrus (Williams et al., 1994). Consequently, most of the recordings from interneurons in this study were from cells with morphology similar to NGF cells. Most, if not all NGF neurons in the hippocampus are known to express NPY. Silencing of NPY expressing interneurons in CA1 by the inhibitory optogenetic protein Jaws resulted in significant reduction of IPSC amplitudes in CA1 PCs. Therefore, these data suggest that NPY-expressing NGFs in the CA1 SLM significantly contribute to feed-forward inhibition of CA1 PCs. Although NGF interneurons appear to play an important role in mediating RE responses in hippocampal CA1, a higher proportion of calretinin-positive interneurons responded to RE input optogenetic stimulation. Previous research showed that calretinin-expressing interneurons preferentially innervate other interneurons (Gulyás et al., 1996). These interneurons are present in all layers of the hippocampus and are known to form extensive dendro-dendritic and axo-dendritic connections with other interneurons (Gulyás et al., 1996). Our results show that stimulation of RE inputs cause robust recruitment of these IS interneurons that is likely to contribute to disinhibition of CA1 PCs (Pi et al., 2013). Thus, RE input onto hippocampal CA1 interneurons will likely have a complex effect involving both inhibitory and disinhibitory circuits. Taken together, data presented in the present study provide novel information regarding the physiological impact of RE stimulation on diverse cell types in hippocampal CA1 and highlights the role of local interneurons in modulation of CA1 function.

## Data Availability Statement

The raw data supporting the conclusion of this article will be made available by the authors, without undue reservation.

## Ethics Statement

The animal study was reviewed and approved by VCU IACUC.

## Author Contributions

PG and AM conceived of the study, formulated the experimental design, and analyzed the data. PG and EL performed the intracranial injections, histology, and neuronal reconstructions. PG performed the electrophysiological experiments and wrote the first draft. EL and AM edited the first draft. All authors contributed to the article and approved the submitted version.

## Conflict of Interest

The authors declare that the research was conducted in the absence of any commercial or financial relationships that could be construed as a potential conflict of interest.

## Publisher’s Note

All claims expressed in this article are solely those of the authors and do not necessarily represent those of their affiliated organizations, or those of the publisher, the editors and the reviewers. Any product that may be evaluated in this article, or claim that may be made by its manufacturer, is not guaranteed or endorsed by the publisher.

## Supplementary Material

Supplementary Figure 1 Glutamate release *via* optogenetic and electrical stimulation have similar short-term kinetic properties. **(A)** Representative current traces obtained from the same CA1 PC in response to 10 × 20 Hz optogenetic stimulation of oChIEF (blue) or electrical stimulation (red) of the Schaffer collateral inputs. **(B)** Line-plot of summary data (*n* = 4 cells) showing that optogenetic and electrical stimulation evokes glutamate release with similar short-term kinetic properties. Amplitudes are normalized to the EPSC response of the first stimulus pulse.
