# Supplementary figures and images for: Nucleus Reuniens Afferents in Hippocampus Modulate CA1 Network Function via Monosynaptic Excitation and Polysynaptic Inhibition
Source: Front Cell Neurosci. 2021 Oct 12;15:660897. doi: 10.3389/fncel.2021.660897 (PMC8545856; doi:10.3389/fncel.2021.660897)

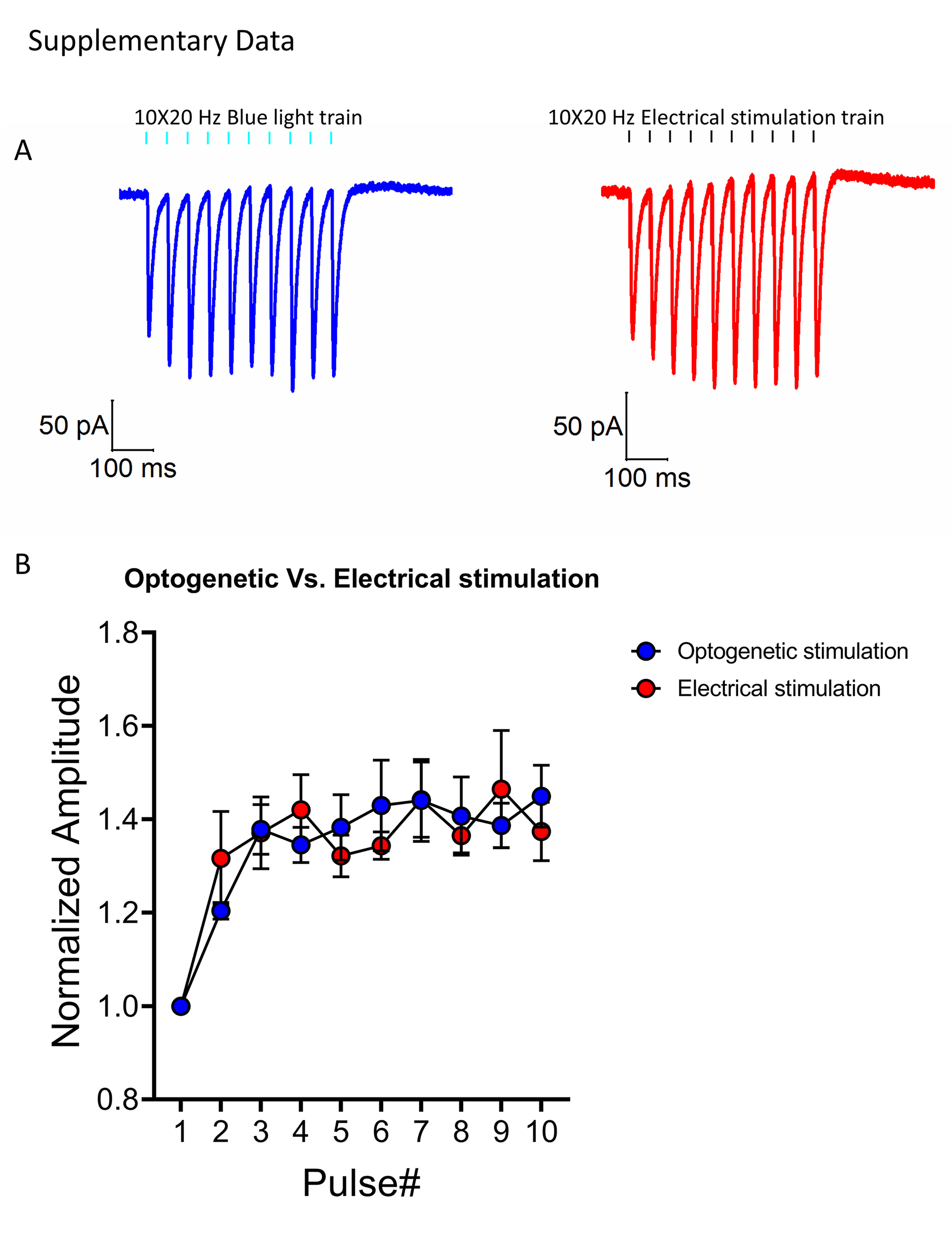

Supplement: Supplementary Figure 1 — Glutamate release via optogenetic and electrical stimulation have similar short-term kinetic properties. (A) Representative current traces obtained from the same CA1 PC in response to 10 × 20 Hz optogenetic stimulation of oChIEF (blue) or electrical stimulation (red) of the Schaffer collateral inputs. (B) Line-plot of summary data (n = 4 cells) showing that optogenetic and electrical stimulation evokes glutamate release with similar short-term kinetic properties. Amplitudes are normalized to the EPSC response of the first stimulus pulse. [file Image_1.TIF]
